# Response to Cysteamine in Osteoclasts Obtained from Patients with Nephropathic Cystinosis: A Genotype/Phenotype Correlation

**DOI:** 10.3390/cells10092498

**Published:** 2021-09-21

**Authors:** Thomas Quinaux, Aurélia Bertholet-Thomas, Aude Servais, Olivia Boyer, Isabelle Vrillon, Julien Hogan, Sandrine Lemoine, Ségolène Gaillard, Candide Alioli, Sophie Vasseur, Cécile Acquaviva, Olivier Peyruchaud, Irma Machuca-Gayet, Justine Bacchetta

**Affiliations:** 1 Centre de Référence des Maladies Rénales Rares, Centre de Référence des Maladies Rares du Calcium et du Phosphore, Filières de Santé Maladies Rares OSCAR, ORKID et ERK-Net, Hôpital Femme Mère Enfant, 69500 Bron, France; itommy640@gmail.com (T.Q.); aurelia.bertholet-thomas@chu-lyon.fr (A.B.-T.); 2 INSERM 1033 Research Unit, 69008 Lyon, France; candide.alioli@inserm.fr (C.A.); olivier.peyruchaud@inserm.fr (O.P.); irma.machuca-gayet@inserm.fr (I.M.-G.); 3 Service de Néphrologie, Hôpital Necker, 75015 Paris, France; aude.servais@aphp.fr; 4 Service de Néphrologie Pédiatrique, Centre de Référence des Maladies Rénales Héréditaires de l’Enfant et de l’Adulte, Hôpital Necker-Enfants Malades, AP-HP, 75015 Paris, France; olivia.boyer@aphp.fr; 5 Service de Néphrologie Dialyse et Transplantation Pédiatriques, Hôpital d’Enfants, CHRU de Nancy, 54000 Nancy, France; i.vrillon@chru-nancy.fr; 6 Service de Néphrologie Pédiatrique, APHP, Hôpital Robert Debré, 75019 Paris, France; julien.hogan@aphp.fr; 7 Service de Néphrologie, Dialyse et Hypertension Artérielle, Hôpital Edouard Herriot, 69008 Lyon, France; sandrine.lemoine01@chu-lyon.fr; 8 Faculté de Médecine Lyon Est, Université de Lyon, 69008 Lyon, France; 9 EPICIME-CIC 1407, Département d’Epidémiologie Clinique, Groupement Hospitalier Est, Hospices Civils de Lyon, 69500 Bron, France; segolene.gaillard@chu-lyon.fr; 10 Service de Biochimie et Biologie Moléculaire, Unité Maladies Héréditaires du Métabolisme, Groupement Hospitalier Est, Hospices Civils de Lyon, 69500 Bron, France; sophie.vasseur@chu-lyon.fr (S.V.); cecile.acquaviva-bourdain@chu-lyon.fr (C.A.)

**Keywords:** cystinosis, cysteamine, bone, osteoclast, genotype

## Abstract

Bone complications of cystinosis have been recently described. The main objectives of this paper were to determine in vitro the impact of CTNS mutations and cysteamine therapy on human osteoclasts and to carry out a genotype-phenotype analysis related to osteoclastic differentiation. Human osteoclasts were differentiated from peripheral blood mononuclear cells (PBMCs) and were treated with increasing doses of cysteamine (0, 50, 200 µM) and then assessed for osteoclastic differentiation. Results are presented as median (min-max). A total of 17 patients (mainly pediatric) were included, at a median age of 14 (2–61) years, and a eGFR of 64 (23–149) mL/min/1.73 m^2^. Most patients (71%) were under conservative kidney management (CKM). The others were kidney transplant recipients. Three functional groups were distinguished for CTNS mutations: cystinosin variant with residual cystin efflux activity (RA, residual activity), inactive cystinosin variant (IP, inactive protein), and absent protein (AP). PBMCs from patients with residual cystinosin activity generate significantly less osteoclasts than those obtained from patients of the other groups. In all groups, cysteamine exerts an inhibitory effect on osteoclastic differentiation at high doses. This study highlights a link between genotype and osteoclastic differentiation, as well as a significant impact of cysteamine therapy on this process in humans.

## 1. Introduction

Nephropathic cystinosis (NC; 1/200,000 live births) is a monogenic autosomal recessive lysosomal storage disease caused by a bi-allelic mutation of the *CTNS* gene (17p13.2), consisting of 12 exons [1]. This gene encodes cystinosin, a lysosomal seven-transmembrane domain cystine transporter of 367 amino acids. So far, over 140 pathogenic mutations have been reported in the *CTNS* gene [2], the most frequent one being a large deletion of 57 kb involving the promoter region and the first 9 exons and part of exon 10. This deletion represents approximately 50% of mutant alleles in patients of North European and North American origin [2].

Cystinosin deficiency causes an accumulation of cystine in all organs and tissues, making cystinosis a systemic disease [3,4]. Early clinical manifestations are related to complete proximal tubulopathy and therefore include polyuric-polydipsic syndrome, growth retardation, and hypophosphatemic rickets. The natural history of this disorder is marked by chronic interstitial nephritis, leading to end stage renal disease during the second decade of life. In this regard, the beneficial role of cysteamine therapy in NC has been well known for nearly four decades: Although it does not prevent nor improve tubulopathy, it considerably slows the progression of renal lesions [4,5], delays the need for transplantation [6], and prevents late complications [7].

Since patient survival has improved considerably with cysteamine therapy, late onset complications have emerged, notably bone involvement. Indeed, the concept of “cystinosis metabolic bone disease” (CMBD) is currently emerging [8]. We were the first to show in a pilot study on 10 teenagers and young adults, at a median age of 23 (range 10–35) years, that 70% of patients complained of a bone symptom (past of fracture, bone deformations, and/or bone pain), with a tendency toward low PTH and low FGF23 levels [9]. At the same time, an American study in 30 patients displayed similar results [10]. Physicians are currently aware of this specific “novel” complication of NC, and international guidelines on the diagnosis and management of CMBD were published in 2019 [8].

Even though its exact underlying pathophysiology remains unclear, at least five distinct but complementary entities can explain CMBD in addition to the classical mineral and bone disorders associated with CKD and post-transplant [8,11]: long-term consequences of hypophosphatemic rickets and renal Fanconi syndrome; deficiency in nutrition and micro-nutrition, and notably copper deficiency; hormonal disturbances such as hypothyroidism, hypogonadism, hypoparathyroidism and resistance to growth hormone and IGF1; myopathy; and intrinsic and iatrogenic bone lesions such as direct bone effects of *CTNS* mutation on osteoblasts and osteoclasts, both in murine models of cystinosis and cystinosis patients [11]. Recent experimental data corroborate clinical observations suggesting a toxicity of high-dose cysteamine on bone cells [12,13,14]. We previously showed that if monocytes derived from NC patients PBMCs were more prone to differentiate into osteoclasts than healthy donor monocytes, they displayed less efficient resorption activity. However, intriguingly enough, cysteamine treatment did not revert this tendency nor did it revert the deficient resorption activity in vitro of NC patients-derived osteoclasts [14]. These findings suggested that cystinosin might be a negative regulator of osteoclast differentiation but also that cystine efflux is not essential to osteoclastogenesis.

Thus, the objectives of the present study are to determine in vitro the impact of *CTNS* mutations and cysteamine therapy on human osteoclasts derived from peripheral blood mononuclear cells (PBMCs), and to carry out a genotype-phenotype analysis in terms of osteoclastic differentiation and response to cysteamine therapy, to better decipher the functional role of cystinosin in osteoclasts.

## 2. Materials and Methods

### 2.1. Clinical Study

The CYSTEABONE study (NCT03919981) was a prospective multicenter clinical study. The main objective of this clinical study was to evaluate the impact of cysteamine on osteoclastic differentiation in patients with NC, depending on the underlying genotype. Inclusion criteria were the following: patients older than 2 years of age, confirmed diagnosis of NC, and ongoing oral cysteamine therapy at inclusion. In addition to the routine biological follow-up, we obtained a sample of total blood in order to conduct osteoclastic differentiation analyses.

Clinical data were recorded: current age; age at diagnosis; renal status (conservative kidney management, CKM, dialysis, renal transplant); date(s) of dialysis initiation/renal transplantation(s); body weight and height; daily dose of cysteamine (keeping in mind that it is usually admitted that the daily dose with delayed-release (DR) cysteamine is around 75% of that using short-acting(SA) cysteamine) [15]; current treatment with growth hormone (rhGH); type of immunosuppression if any; and characterization of genetic mutation(s) and clinical bone symptoms, i.e., history of fracture(s), bone pain, bone deformities, and details concerning orthopedic surgery. Routine biological data were also recorded: plasma creatinine and estimated glomerular filtration rate (eGFR) using the 2009 Schwartz formula [16], calcium, phosphate (expressed as SDS for age) [17], and total alkaline phosphatase (ALP) expressed as xx-fold the upper normal limit of ALP for age and gender [18], parathyroid hormone (PTH) and 25-hydroxyvitamin D levels, as well as an average concentration of white blood cell hemicystin concentration in the past year. Since techniques for hemicystin were different among centers, we presented the results from the two different assays separately.

### 2.2. Primary Cultures of Human Osteoclasts

Blood samples were drawn fasting before the administration of cysteamine, whose plasmatic concentration was therefore residual in patients receiving maintenance cysteamine therapy. As previously published [14,19], mononuclear cells were purified from peripheral blood, loaded onto a lymphocyte separation medium (Eurobio, Courtaboeuf, France), fractionated in a density gradient in order to purify peripheral blood mononuclear cells (PBMCs) and then seeded in 96-wells plates. Osteoclasts were obtained by incubating PBMCs with M-CSF at 20 ng/mL (PeproTech, Rocky Hill, NJ, USA) and RANKL at 40 ng/mL (PeproTech) from day 1 to terminal differentiation. By day 3, osteoclast precursors were treated with increasing doses of cysteamine during differentiation: 0 (baseline conditions), 50, and 200 μM. At the end of the osteoclastic differentiation protocol, cells were collected in Trizol reagent (Invitrogen, Thermo Fisher Scientific, Waltham, MA, USA) for real-time PCR analysis or fixed with 4% paraformaldehyde (PFA) and submitted to histochemical staining using a TRAP staining kit, in accordance with the manufacturer’s instructions (Sigma-Aldrich, St. Louis, MO, USA). Positively labeled cells with over three nuclei were then counted to assess in vitro osteoclastic differentiation of PBMCs.

### 2.3. Statistical Analyses

Clinical and biological data in patients are presented as median (min-max). Comparison between groups was performed using a Chi-square test or non-parametric Mann–Whitney tests. Data concerning osteoclastic differentiation are presented as mean number of osteoclasts per well ± standard error of the mean (SEM). Values collected under different cell culture conditions were compared using one-way analysis of variance (ANOVA) followed by Bonferroni’s post hoc test for multiple comparisons. Data regarding osteoclastic differentiation of mononuclear progenitors according to their genotype, at various cysteamine concentrations, were compared using the Mann–Whitney test. A result with *p* < 0.05 was considered significant. Analyses were performed using the PRISM 5 software.

### 2.4. Ethical Considerations

The CYSTEABONE study was approved by the *Comité de Protection des Personnes Sud-Méditerranée IV* (2019-A00166-51). All patients and/or parents gave informed oral consent (*Jardé type 3 protocol* by French law).

## 3. Results

### 3.1. Patients’ Clinical Characteristics

Seventeen patients suffering from NC, of which nine females, were included in this study, from different tertiary centres in France (four pediatric and two adult units). Most subjects were pediatric patients, with a median age of 14 (2–61) years, and eGFR of 64 (23–149) mL/min per 1.73 m^2^. Most patients (71%) were undergoing CKM, and five of them had received a kidney transplant (29%). Baseline characteristics of the patients, including details regarding mutations in the *CTNS* gene and involved exons, are summarized in Table 1.

In total, 47% of patients displayed bone symptoms, and 17% had to undergo orthopedic surgery. Patient 6 presented a spontaneous fracture of the metatarsal bone at the age of 16, and patient 15 presented a trauma fracture of the metacarpal bone at the age of 58. Among the seven patients who displayed bone deformations, there were three scoliosis/kyphosis, two pectus carinatum, and six genu valgum/varum. Surgery was performed in 38% of patients presenting with overt bone symptoms. Therapeutic compliance was rather satisfactory in the cohort, since only five patients out of 17 displayed hemicystin levels above the local target. As expected, the median daily doses of cysteamine were lower in patients receiving DR cysteamine as compared to the ones receiving SA cysteamine: 1012(368–1902) and 1632(1236–3607) mg/m^2^ (*p* = 0.003). Table 2 compares these two-sub-groups, the only significant difference being the proportion of patients within the target for LHL.

We also distinguished patients according to their renal status: CKM or renal transplant, as illustrated in Table 3. Patients receiving CKM were significantly younger than transplant recipients. In these young patients, bone symptoms of any kind were significantly less common than in older transplant patients but already present (33% versus 80% respectively, *p* = 0.04), although eGFR did not differ significantly. The number of osteoclasts obtained at the end of the differentiation process did not differ among the two groups.

### 3.2. High Dose Cysteamine Decreases the Propensity of Patients-Derived Mononuclear Progenitors to Generate Osteoclasts

**Figure 1:**
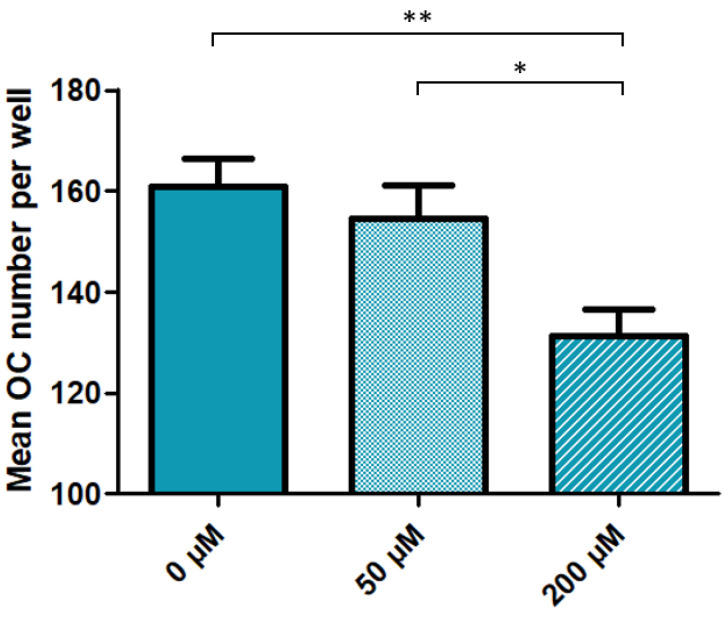
Impact of cysteamine treatment on osteoclast differentiation in patients with nephropathic cystinosis. Osteoclasts (TRAP-positive cells with over three nuclei) were generated from PBMCs of NC patients and treated with increasing doses of cysteamine (untreated, 50 and 200 μM). Results in terms of osteoclasts number are presented as means for seven to eight wells, with SEM. A total of 13 patients were included in the analysis, as results of cell cultures were not satisfactory for the remaining four patients. OC, osteoclasts; TRAP, Tartrate-Resistant Acid Phosphatase; PBMC, Peripheral Blood Mononuclear Cells; SEM, Standard Error of the Mean. * *p* < 0.05, and ** *p* < 0.01 compared between indicated groups by Anova followed by Bonferroni.

Results of in vitro osteoclastic differentiation with different cysteamine concentrations for each patient are summarized in Table 4: Except for two patients, a decreased number of TRAP positive multinucleated cells was observed with high doses of cysteamine (200 µM), as compared to baseline conditions and low doses of cysteamine (50 µM). This decrease was overall significant, as illustrated in Figure 1. Importantly, all osteoclastic cultures were generated from the same number of monocyte progenitors at the beginning of the experiment. Thus, these findings indicate that, at high concentrations, cysteamine decreases the propensity of patients-derived mononuclear progenitors to generate osteoclasts.

### 3.3. Inactive or Absent Cystinosin in Monocyte-Macrophage Precursors Favor Osteoclast Formation Whereas Cysteamine Treatment Impairs It Independently of the Genotype

**Figure 2:**
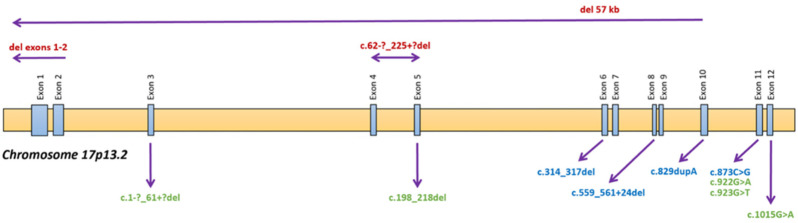
Topography of the mutations in the CTNS gene in this cohort. Schematic illustration of the CTNS gene with display of the mutations’ genomic location within our cohort. Exonic mutations are displayed in the bottom area of the figure. Large deletions are displayed in the top area. Green: residual activity (RA); Blue: inactive protein (IP); Red: absent protein (AP).

Patients were divided into three groups according to the impact of their mutations on the translated cystinosin. Indeed, we distinguished three functional groups: those that led to the synthesis of a cystinosin variant with residual cystin efflux activity (RA, residual activity), those that led to the synthesis of an inactive cystinosin variant (IP, inactive protein), and those that did not allow for the protein to be translated and present at the lysosome membrane (AP, absent protein). The justification of mutations classification is proposed in Table 5 [2,20,21,22,23,24]. The localization of the different mutations is illustrated in Figure 2 [1,2,20,23,25,26].

Sub-group analyses depending on the expected cystinosin functionality were also performed from a clinical and biochemical point of view, as illustrated in Table 6: Even though statistical significance was not obtained, the AP sub-group seemed to be less well controlled in terms of hemicystin levels, and the RA sub-group appeared to be older than the other sub-groups. However, Spearman bivariate analyses showed no significant association between age and the number of obtained osteoclasts at the end of the differentiation process (−R = −0.424, *p* = NS).

**Figure 3:**
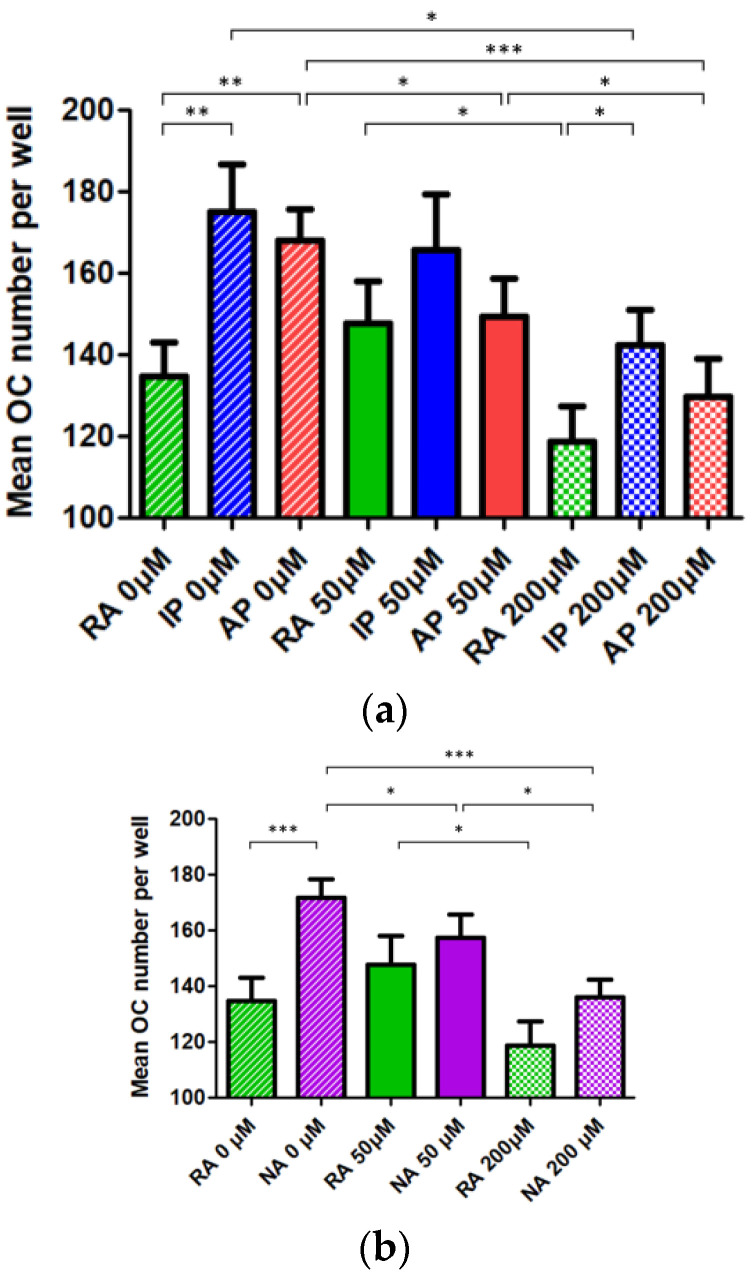
Impact of genotype and cysteamine treatment on osteoclast differentiation in patients with nephropathic cystinosis, (**a**) when analyzing the three genotypes independently, and (**b**) when combining the inactive and absent protein. Osteoclasts (TRAP-positive cells with over three nuclei) were generated from PBMCs of cystinotic patients, and treated with increasing doses of cysteamine (untreated, 50 and 200 μM). Results in terms of osteoclasts number are presented as means for seven to eight wells, with SEM. A total of 11 patients were included in the analysis, as data on genotype were not available for two of the 13 patients whose cultures developed properly. RA, residual activity; IP, inactive protein; AP, absent protein; NA: inactive or absent protein. OC, osteoclasts; TRAP, Tartrate-Resistant Acid Phosphatase; PBMC, Peripheral Blood Mononuclear Cells; SEM, Standard Error of the Mean. * *p* < 0.05, ** *p* < 0.01 and *** *p* < 0.001 compared between indicated groups.

We therefore performed a genotype/phenotype analysis, the read-out being osteoclastic differentiation of monocyte progenitors from patients with nephropathic cystinosis depending on the underlying genotype, as illustrated in Figure 3a. At baseline, the number of osteoclasts was significantly higher in the IP and AP groups than it was in the RA group. In these two later groups (IP and AP), the number of osteoclasts obtained in cultures dropped alongside with the increase in cysteamine concentration, in a dose-dependent manner, although it was only statistically significant at high doses. In the RA group, there was no difference in the mean osteoclast number per well without and with 50 µM of cysteamine, in contrast with a significant decrease at 200 µM (as compared to 50 µM but not with absence of cysteamine). Overall for each of the three groups, treatment with a moderate dose of cysteamine (50µM) had no or mild effect, indicating that cystine efflux is likely not involved in the process of osteoclast formation. On the other hand, the decrease in osteoclast number at 200 µM of cysteamine appeared less pronounced in the RA group when compared to pooled IP and AP results as shown in Figure 3b.

## 4. Discussion

Mineral and bone homeostasis disorders displayed by CKD patients increase as kidney function declines. It results in a high number fractures and ectopic vascular calcifications as a consequence of impaired mineral metabolism. In nephropathic cystinosis, these aspecific mineral and bone disorders are worsened by what is now called CMBD [8]. From a clinical point of view, we here confirm that bone involvement is a late complication of cystinosis occurring in teenagers and young adults. Indeed, transplant patients were significantly older than patients under conservative management, and they also presented a higher frequency of bone symptoms. Interestingly, even though the study was not designed for this aim, we here show that the only significant difference between patients receiving SA or DR cysteamine is the proportion of patients within the target for LHL. Additionally, the fact that the ratio DR/SA is 0.62, as opposed to 0.75 in previous publications [15], likely reflects a better compliance in patients receiving DR cysteamine, as expected [27]. This is an indirect plea in “real life” for using DR whenever possible, to optimize the control of LHL, even though there were no differences in term of osteoclastic differentiation in these two sub-groups.

The underlying pathophysiology of CMBD nevertheless remains complex and multi-factorial, but cellular defects have been well documented at the cellular level, both in osteoblasts and osteoclasts, in terms of differentiation and specific activity [12,13,14]. However, cystinosin function in bone cells, and particularly in osteoclasts, remains unclear. Here, we focused on osteoclasts in order to better explain the altered bone phenotype of patients with nephropathic cystinosis. The main strength of this study is the protocol implemented to obtain human bone cells directly from patients presenting an orphan disease. It is an innovative, minimally invasive but time-consuming technique that allows direct access to osteoclasts from a small sample of total blood sample.

Thus, we extend the results of our previous work on cystinosin-induced osteoclastic dysfunction, in which we showed that cystinosin is required for proper osteoclastic differentiation with a peak of expression on day 6 of the differentiation process following the same pattern as cathepsin K transcripts [14]. We also showed that cysteamine has anti-resorptive effects in vitro on osteoclasts derived both from controls and patients [14].

Herein, we have classified cystinosin-identified mutations in three groups corresponding to its predicted in vivo activity, in an attempt to assign a phenotype to a genotype. The main findings of the present study are therefore the following: cells with residual cystinosin activity generate less osteoclasts as opposed to inactive or absent protein, indicating that cystinosin might be a negative modulator of osteoclast formation; moderate doses of cysteamine have no effect on either of the three groups, that is to say that in the RA, IP, or AP group, cysteamine treatment did not increase nor further reduce the number of osteoclasts; osteoclast formation remained of the same order of magnitude, supporting our previous results showing no evidence of a significant effect of cysteamine on osteoclastic differentiation at low doses [14]. In contrast, we here demonstrate a significant inhibitory effect of cysteamine on osteoclastic differentiation at higher doses. These apparent discrepancies with our previous results may be explained by an increased number of patients in this study (17 versus 7), but also by the different clinical profiles of the patients, our previous cases being older (median age 31 years), at different stages of kidney disease (transplantation, N = 5, hemodialysis, N = 2, no conservative management) [14]. Anyway, this inhibitory effect of cysteamine on osteoclastic differentiation appeared to be dose-dependent in the pooled IP and AP groups whereas the response profile to cysteamine appeared to be different in the RA group, as the number of osteoclasts at low doses of cysteamine remained comparable to the number of osteoclasts at baseline.

The genotype/phenotype analysis that we propose hinges on the first rational classification of mutations in the *CTNS* gene and is based on the functional consequences of these mutations on the structure of cystinosin. The justification of mutations classification is based on a multi-disciplinary approach taking into account both published experimental data and patients’ clinical phenotype, with a discussion involving physicians, biochemists, geneticists, and basic scientists [2,20,21,22,23]. In light of the results, this classification appears relevant since it makes it possible to predict a response profile to cysteamine as a function of the patient’s underlying genotype. These findings may be of clinical interest for the management of cysteamine therapy, which should reconcile, especially in patients most at risk of toxicity, effectiveness in reducing lysosomal cystine concentrations and preservation of bone capital. Without cysteamine, the number of osteoclasts was higher in cultures from subjects in whom cystinosin was inactive or absent, as compared to subjects in whom cystinosin retained residual activity. One may argue that a potential bias in the interpretation of these results may be induced by the different number of osteoclasts obtained depending on age, since younger healthy donors are more prone to produce more cells, but there was no significant association between age and osteoclastic number at the end of the differentiation process in the cells obtained from these peculiar patients. The fact that all but one patient received maintenance cysteamine therapy may also influence the results of subsequent cell culture experiments, but it would not be ethical to propose a wash-out period in these patients.

Mechanistically, this observation of a different profile of osteoclastogenesis depending on the underlying cystinosin functionality may be explained by the role that the mammalian target of rapamycin complex 1 (mTORC1) and its interaction with the Ragulator–Rag complex play during osteoclastogenesis, as discussed thoroughly in a recent review on the topic [11]. Indeed, it has been established that mTORC1 activity is down-regulated during osteoclastic differentiation through the negative regulator TSC1, whose absence impairs RANKL-dependent osteoclastogenesis. Furthermore, Andrzejewska et al. showed that the mTORC1 pathway is downregulated in proximal tubular cell lines derived from Ctns^−/−^ mice [28]. A similar down-regulation in human osteoclastic progenitors might account for the overall increased osteoclastogenesis that we observe in NC patients, as compared to controls. Andrzejewska et al. also demonstrated that cystinosin is a component of the vacuolar H + -ATPase–Ragulator–Rag complex, which controls mTORC1 localization to lysosomes and thus, mTORC1 signaling [28].

DNA mutations in the *CTNS* gene have various functional consequences linked to their structural impact on cystinosin. Extensive deletions (such as the 57-kb deletion) cause the absence of protein, while severe truncating mutations lead to the synthesis of an inactive variant. Both these situations amount to a loss of cystinosin efflux function. In contrast, milder mutations allow the synthesized cystinosin variant to retain residual activity. It is interesting to hypothesize that, as well as canceling cystinosin efflux function, severe *CTNS* mutations impair the interaction between the Ragulator–Rag complex (of which cystinosin is a component) and mTORC1, preventing its activation. On the other hand, mutations of more limited structural impact might allow, to some extent, to maintain an efflux activity as well as the interaction between mTORC1 and the lysosomal membrane-attached Ragulator–Rag complex. This hypothesis would explain the correlation we observed at baseline between the severity of the mutation, its impact on cystinosin efflux function, and the outcome in terms of osteoclastogenesis (increased osteoclast number in the AP and IP groups, compared to the RA group). It could be argued that the downregulation of mTORC1 is due to the accumulation of cystine, which would logically be greater in the AP and IP groups. However, as shown in the Andrzejewska et al. study, decrease of lysosomal cystine levels by cysteamine did not rescue mTORC1 activation in proximal tubular cells, thus suggesting that the downregulation of mTORC1 is due to the absence of cystinosin rather than to the accumulation of cystine [28].

## 5. Conclusions

Bone involvement is a late complication of nephropathic cystinosis, whose recent description is linked to the considerable improvement in patients’ survival under cysteamine therapy. In regards to its clinical importance and deleterious effects on patients’ quality of life, recent international guidelines on evaluation and management of NC bone disease have been published, but its exact underlying pathophysiology remains to be fully determined.

In addition to its beneficial effects in terms of renal survival and overall morbidity and mortality, cysteamine has a direct effect on bone metabolism, which depends on the concentration at which it is administered. Here, the differences observed in terms of osteoclastic outcomes between the different genotypes confirm that cystinosin has a modulating role on osteoclastogenesis.

## Figures and Tables

**Table 1:** Baseline characteristics of patients.

Pt	Age at Diag (Years)	Renal Status	Age at Eval (Years)	Sex	DNA Mutation	Protein Predicted Effect	Affected Exons	GFR	Cysteamine Daily Dose (mg/m^2^)	Type of Cysteamine	LHL < 1	LHL < 2	rhGH	Past of Fracture	BD	BP	Any Bone Symptoms	Orthopedic Surgery
**1**	1.3	C	4	M	c.922G > A/c.922G > A	RA	11/11	34	564	DR	0.7		No	No	No	No	No	No
**2**	2.5	T	30	F	c.1015G > A/del 57 kb	RA	12/1 to 10	62	1632	SA		2.1	No	No	**Yes**	No	**Yes**	No
**3**	1.7	C	15	F	c.829dupA/c.829dupA	IP	10/10	38	1630	SA		1.9	**Yes**	No	**Yes**	**Yes**	**Yes**	No
**4**	0.9	C	5	F	del 57 kb/del 57 kb	AP	1 to 10/1 to 10	71	954	DR	1		No	No	No	No	No	No
**5**	1.0	C	7	F	del 57 kb/c.1-?_61 + ?del	RA	1 to 10/3	79	580	DR	0.2		No	No	No	No	No	No
**6**	5.5	T	17	M	c.314_317del/c.314_317del	IP	6/6	112	986	DR	0.7		No	**Yes**	**Yes**	**Yes**	**Yes**	No
**7**	0.8	C	16	M	del 57 kb/del 57 kb	AP	1 to 10/1 to 10	23	1236	SA		3.7	**Yes**	No	**Yes**	**Yes**	**Yes**	**Yes**
**8**	1.2	C	3	F	del 57 kb/c.873C > G	IP	1 to 10/11	149	2505	SA		1.8	No	No	No	No	No	No
**9**	0.1	C	2	F	del 57 kb/c.873C > G	IP	1 to 10/11	105	3607	SA		2.2	No	No	No	No	No	No
**10**	4.0	T	18	F	del 57 kb/c.62-?_225 + ?del	AP	1 to 10/4 and 5	105	1457	SA	0.2		No	No	No	No	No	No
**11**	1.0	C	14	F	del 57 kb/c.62-?_225 + ?del	AP	1 to 10/4 and 5	64	1761	SA	1.1		**Yes**	No	No	No	No	No
**12**	2.0	C	9	M	del 57 kb/del 57 kb	AP	1 to 10/1 to 10	84	1420	DR	0.8		**Yes**	No	No	No	No	No
**13**	5.3	C	8	M	c.198_218del/c.559_561 + 24del	RA	5/8	62	1111	DR		1.4	**Yes**	No	**Yes**	No	**Yes**	No
**14**	1.3	C	15	M	del 57 kb/del 57 kb	AP	1 to 10/1 to 10	127	1232	DR		1	**Yes**	No	No	No	No	No
**15**	1.1	T	61	F	del 57 kb/c.923G > T	RA			368 (theory) but 0 in reality	DR	0.7		No	**Yes**	No	No	**Yes**	No
**16**	6.5	T	18	M	Del exons 1–2/del exons 1–2	AP	1 and 2	59	1039	DR		3.3	No	No	**Yes**	**Yes**	**Yes**	**Yes**
**17**	2.0	C	14	M	Del exons 1–2/del exons 1–2	AP	1 and 2	28	1902	DR		1.2	No	No	**Yes**	No	**Yes**	**Yes**

PN, patient number; GFR, glomerular filtration rate (mL/min/1.73 m^2^); rhGH, recombinant human growth hormone; LHL, leukocyte hemicystin levels (µmol/g of proteins), obtained from two different labs and as such presented in different columns with the target value for each lab displayed on top of the table; BD, bone deformation; BP, bone pain; C, conservative kidney management; T, kidney transplant; M, male; F, female; RA, residual activity; IP, inactive protein; AP, absent protein; DR: delayed release cysteamine (PROCYSBI^®^); SA: short acting cysteamine (CYSTAGON^®^); diag: diagnosis; eval: evaluation.

**Table 2:** Patients’ characteristics according to the type of cysteamine.

Nephropathic Cystinosis Patients	Short Acting Cysteamine	Delayed Release Cysteamine
Number of patients	7	10
Age (y/o)	15 (2–30)	12 (4–61)
Cysteamine daily dose (mg/m^2^) *	1632 (1236–3607)	1012 (368–1902)
Patients in the target for LHL *	3 (43%)	9 (90%)
GFR (mL/min per 1.73 m^2^)	46 (16–149)	65 (33–84)
Calcium (mmol/L)	2.27 (2.11–2.50)	2.42 (2.23–2.92)
Phosphate (standard deviation for age)	−1.8 (−4.2;1.7)	−1.5 (−3.6;2.4)
PTH (ng/L)	34 (18–127)	20 (5–90)
25-D (ng/mL)	28 (10–42)	26 (21–49)
Total ALP (times the upper physiological value for gender and age)	0.87 (0.41–4.29)	0.74 (0.28–1.19)
Any bone symptoms (%)	3 (43%)	5 (50%)
Number of osteoclasts obtained at the end of the differentiation process	168 (97–187)	162 (111–203)

Results are presented as median (min-max) and percentage. * *p* < 0.05; LHL, leukocyte hemicystin levels; GFR: glomerular filtration rate; PTH: parathyroid hormone; 25-D: 25 hydroxy vitamin D; ALP: alkaline phosphatase.

**Table 3:** Patients’ characteristics according to renal management modality.

Nephropathic Cystinosis Patients	Conservative Management	Renal Transplantation
Number of patients	12	5
Patients receiving SA cysteamine	5	2
Age (y/o) *	9 (2–16)	18 (17–61)
Cysteamine daily dose (mg/m^2^)	1328 (564–3607)	1039 (368–1632)
Patients in the target for LHL	9 (75%)	3 (60%)
GFR (mL/min per 1.73 m^2^)	65 (16–149)	56 (45–76)
Calcium (mmol/L)	2.40 (2.23–2.92)	2.42 (2.11–2.57)
Phosphate (standard deviation for age)	−1.6 (−4.2;2.4)	−1.4 (−2.8;−0.5)
PTH (ng/L)	21 (8–90)	32 (5–127)
25-D (ng/mL)	28 (10–49)	26 (22–26)
Total ALP (times the upper physiological value for gender and age)	0.9 (0.3–4.2)	0.5 (0.4–0.8)
Any bone symptoms (%) *	33	80
Number of osteoclasts obtained at the end of the differentiation process	159 (94–203)	165 (105–181)

Results are presented as median(min-max) and percentage. * *p* < 0.05; SA: short-acting; LHL, leukocyte hemicystin levels; GFR: glomerular filtration rate; PTH: parathyroid hormone; 25-D: 25 hydroxy vitamin D; ALP: alkaline phosphatase.

**Table 4:** Osteoclastic differentiation outcomes in each patient.

Patient Number	Protein Functionality	Cysteamine Concentration		
		0 µM	50 µM	200 µM
1	RA	203 ± 41	185 ± 44	147 ± 31
2	RA	105 ± 11	122 ± 13	67 ± 6
5	RA	111 ± 12	121 ± 19	97 ± 14
13	RA	117 ± 7	125 ± 12	107 ± 9
15	RA	197 ± 9	210 ± 20	178 ± 8
*3*	*IP*	*44 ± 4*	*36 ± 5*	*29 ± 3*
6	IP	181 ± 14	183 ± 23	147 ± 14
8	IP	171 ± 16	180 ± 13	160 ± 13
9	IP	187 ± 19	206 ± 22	205 ± 25
*4*	*AP*	*65 ± 7*	*75 ± 6*	*52 ± 7*
7	AP	94 ± 11	90 ± 6	100 ± 6
10	AP	168 ± 14	173 ± 17	127 ± 5
*11*	*AP*	*48 ± 3*	*48 ± 4*	*42 ± 5*
12	AP	148 ± 9	115 ± 9	87 ± 6
*14*	*AP*	*44 ± 5*	*48 ± 4*	*39 ± 3*
16	AP	162 ± 16	129 ± 17	114 ± 14
17	AP	172 ± 20	121 ± 14	105 ± 10

Multinucleated TRAP-positive cells (over three nuclei) were generated from PBMCs with increasing doses of cysteamine (untreated, 50 and 200 μM) and counted. RA, Residual Activity; IP, Inactive Protein; AP, Absent Protein; The results are presented as means for 7 to 8 wells ± SEM (standard error of the mean). In italics, the results of differentiation were not taken into account for Figures 1 and 3, and Tables 2 and 3, because of the low number of obtained cells that may impact the global results.

**Table 5:** Justification of the classification of the mutations.

	DNA Mutation	Protein Mutation	Protein Predicted Effect	Justification Based on Experimental Data and Clinical Phenotype
**1**	c.922G > A/c.922G > A	p.G308R/p.G308R	RA	Clinically quite severe (advanced CKD at 4 years of age) despite early diagnosis and satisfactory compliance, quite low and stable cysteamine doses with LHL within the target, in experimental models prediction of abolished transport [20].
**2**	c.1015G > A/del 57 kb	p.G339R/p.?	RA	Heterozygous form of the large deletion of CTNS + point mutation on the last exon. Transplantation at the age of 11 years in 2000 (median age at that time for transplantation in historical cohorts), standard cysteamine daily dose, and prediction of severe impact (but no functional analysis of transport) [21]. Point mutation in the last transmembrane domain in the C-terminal part may be important for protein–protein interaction.
**3**	c.829dupA/c.829dupA	p.T277NfsX19/p.T277NfsX19	IP	Premature stop [21]
**4**	del 57 kb/del 57 kb	p.?/p.?	AP	Homozygous form of the large deletion of CTNS
**5**	del 57 kb/c.1-?_61 + ?del	p.?/p.?	RA	Heterozygous form of the large deletion of CTNS + deletion exon 3 (first coding exon). This second mutation was never described. Is there an alternative start? Clinically stable, satisfactory compliance, and quite low and stable cysteamine doses with LHL within the target.
**6**	c.314_317del/c.314_317del	p.H105PfsX12/p.H105PfsX12	IP	Stop in exon 6, this second mutation was not described.
**7**	del 57 kb/del 57 kb	p.?/p.?	AP	Homozygous form of the large deletion of CTNS
**8**	del 57 kb/c.873C > G	p.?/p.Tyr291X	IP	Heterozygous form of the large deletion of CTNS + early stop in exon 11. Severe clinical phenotype.
**9**	del 57 kb/c.873C > G	p.?/p.Tyr291X	IP	Heterozygous form of the large deletion of CTNS + early stop in exon 11. Severe clinical phenotype.
**10**	del 57 kb/c.62-?_225 + ?del	p.?/p.?	AP	Heterozygous form of the large deletion of CTNS + exons 4 and 5 missing at the beginning of the protein. This second mutation was never described.
**11**	del 57 kb/c.62-?_225 + ?del	p.?/p.?	AP	Heterozygous form of the large deletion of CTNS + exons 4 and 5 missing at the beginning of the protein. This second mutation was never described.
**12**	del 57 kb/del 57 kb	p.?/p.?	AP	Homozygous form of the large deletion of CTNS.
**13**	c.198_218del/c.559_561 + 24del	p.Ile67_Pro73del/splicing	RA	Clinically stable, satisfactory compliance, and quite low and stable cysteamine doses with LHL within the target. The first mutation is described with residual activity [22]. The second mutation induces a splicing and leads to a truncated protein [24].
**14**	del 57 kb/del 57 kb	p.?/p.?	AP	Homozygous form of the large deletion of CTNS.
**15**	del 57 kb/c.923G > T	p.?/p.G308V	RA	Diagnosis at 13 months, ESRD 14 years, transplantation 18 years, still on the first graft, bad compliance, two pregnancies. Initiation of CYSTAGON at 37 years of age, switch to PROCYSBI at the age of 60 years. Very atypical clinical course with mild phenotype. Moreover, the described functional impact of the second mutation favors the existence of residual activity [23].
**16**	Del exons 1–2/del exons 1–2	p.?/p.?	AP	Severe clinical phenotype with muscular impairment. Likely corresponds to the homozygous form of the large CTNS deletion. Could correspond to a contiguous gene syndrome.
**17**	Del exons 1–2/del exons 1–2	p.?/p.?	AP	Severe clinical phenotype. Likely corresponds to the homozygous form of the large CTNS deletion. Could correspond to a contiguous gene syndrome.

Protein mutation “p.?”, protein variant of undetermined structure; fs frameshift; del: deletion; LHL, leukocyte hemicystin levels, CKD: chronic kidney disease.

**Table 6:** Patients’ characteristics according to the underlying genotype.

Nephropathic Cystinosis Patients	RA	IP	AP
Number of patients	5	4	8
Age (y/o)	22 (4;61)	9 (2;17)	14 (5;18)
CKM/ Tx (N/N)	3/2	3/1	6/2
Past of rhGH therapy (N)	1	1	4
Cysteamine daily dose (mg/m^2^) *	777 (0;1632)	1932 (986;3607)	1375 (954;1902)
Number of patients receiving SA cysteamine	1 (20%)	3 (75%)	3 (38%)
Proportion of patients in the target for LHL	4 (80%)	3 (75%)	5 (62%)
Number of patients with past of rhGH	1	1	4
GFR (ml/min per 1.73 m^2^)	57 (34;79)	101 (38;149)	70 (23;127)
Calcium (mmol/L)	2.5 (2.1;2.9)	2.5 (2.4;2.5)	2.3 (2.2;2.6)
Phosphate (standard deviation for age)	−1.4 (−3.6;2.4)	−1.9 (−2.9;−1.2)	−1.3 (−4.2;1.7)
PTH (ng/L)	35 (8;127)	22 (18;37)	36 (5;90)
25-D (ng/mL)	28 (22;35)	30 (26;39)	28 (10;49)
Total ALP (times the upper physiological value for gender and age)	0.7 (0.3;1.2)	0.7 (0.4;0.9)	1.5 (0.4;4.3)
Any bone symptoms	60%	50%	38%

Results are presented as median (min-max) and percentage; * *p* < 0.05; CKM: conservative kidney management/Tx: past of renal transplantation; SA: short-acting; LHL, leukocyte hemicystin levels; GFR: glomerular filtration rate; PTH: parathyroid hormone; 25-D: 25 hydroxy vitamin D; ALP: alkaline phosphatase.

## Data Availability

Datasets analyzed during the current study are not publicly available but are available from the corresponding author on reasonable request.

## References

[1] Town M., Jean G., Cherqui S., Attard M., Forestier L., Whitmore S.A., Callen D.F., Gribouval O., Broyer M., Bates G.P. (1998). A novel gene encoding an integral membrane protein is mutated in nephropathic cystinosis. Nat. Genet..

[2] David D., Princiero Berlingerio S., Elmonem M.A., Oliveira Arcolino F., Soliman N., van den Heuvel B., Gijsbers R., Levtchenko E. (2019). Molecular Basis of Cystinosis: Geographic Distribution, Functional Consequences of Mutations in the CTNS Gene, and Potential for Repair. Nephron.

[3] Gahl W.A., Thoene J.G., Schneider J.A. (2002). Cystinosis. N. Engl. J. Med..

[4] Emma F., Nesterova G., Langman C., Labbé A., Cherqui S., Goodyer P., Janssen M.C., Greco M., Topaloglu R., Elenberg E. (2014). Nephropathic cystinosis: An international consensus document. Nephrol. Dial. Transplant..

[5] Markello T.C., Bernardini I.M., Gahl W.A. (1993). Improved renal function in children with cystinosis treated with cysteamine. N. Engl. J. Med..

[6] Brodin-Sartorius A., Tête M.-J., Niaudet P., Antignac C., Guest G., Ottolenghi C., Charbit M., Moyse D., Legendre C., Lesavre P. (2012). Cysteamine therapy delays the progression of nephropathic cystinosis in late adolescents and adults. Kidney Int..

[7] Gahl W.A., Balog J.Z., Kleta R. (2007). Nephropathic cystinosis in adults: Natural history and effects of oral cysteamine therapy. Ann. Intern. Med..

[8] Hohenfellner K., Rauch F., Ariceta G., Awan A., Bacchetta J., Bergmann C., Bechtold S., Cassidy N., Deschenes G., Elenberg E. (2019). Management of bone disease in cystinosis: Statement from an international conference. J. Inherit. Metab. Dis..

[9] Bertholet-Thomas A., Claramunt-Taberner D., Gaillard S., Deschênes G., Sornay-Rendu E., Szulc P., Cohen-Solal M., Pelletier S., Carlier M.-C., Cochat P. (2018). Teenagers and young adults with nephropathic cystinosis display significant bone disease and cortical impairment. Pediatr. Nephrol..

[10] Florenzano P., Ferreira C., Nesterova G., Roberts M.S., Tella S.H., de Castro L.F., Brown S.M., Whitaker A., Pereira R.C., Bulas D. (2018). Skeletal Consequences of Nephropathic Cystinosis. J. Bone Miner. Res..

[11] Machuca-Gayet I., Quinaux T., Bertholet-Thomas A., Gaillard S., Claramunt-Taberner D., Acquaviva-Bourdain C., Bacchetta J. (2020). Bone Disease in Nephropathic Cystinosis: Beyond Renal Osteodystrophy. Int. J. Mol. Sci..

[12] Conforti A., Taranta A., Biagini S., Starc N., Pitisci A., Bellomo F., Cirillo V., Locatelli F., Bernardo M.E., Emma F. (2015). Cysteamine treatment restores the in vitro ability to differentiate along the osteoblastic lineage of mesenchymal stromal cells isolated from bone marrow of a cystinotic patient. J. Transl. Med..

[13] Battafarano G., Rossi M., Rega L.R., Di Giovamberardino G., Pastore A., D’Agostini M., Porzio O., Nevo N., Emma F., Taranta A. (2019). Intrinsic Bone Defects in Cystinotic Mice. Am. J. Pathol..

[14] Claramunt-Taberner D., Flammier S., Gaillard S., Cochat P., Peyruchaud O., Machuca-Gayet I., Bacchetta J. (2017). Bone disease in nephropathic cystinosis is related to cystinosin-induced osteoclastic dysfunction. Nephrol. Dial. Transplant..

[15] Ahlenstiel-Grunow T., Kanzelmeyer N.K., Froede K., Kreuzer M., Drube J., Lerch C., Pape L. (2017). Switching from immediate- to extended-release cysteamine in nephropathic cystinosis patients: A retrospective real-life single-center study. Pediatr. Nephrol..

[16] Schwartz G.J., Work D.F. (2009). Measurement and estimation of GFR in children and adolescents. Clin. J. Am. Soc. Nephrol..

[17] Ardeshirpour L., Cole D.E.C., Carpenter T.O. (2007). Evaluation of bone and mineral disorders. Pediatr. Endocrinol Rev..

[18] Shaw J.L.V., Cohen A., Konforte D., Binesh-Marvasti T., Colantonio D.A., Adeli K. (2014). Validity of establishing pediatric reference intervals based on hospital patient data: A comparison of the modified Hoffmann approach to CALIPER reference intervals obtained in healthy children. Clin. Biochem..

[19] Bernardor J., Flammier S., Ranchin B., Gaillard S., Platel D., Peyruchaud O., Machuca-Gayet I., Bacchetta J. (2020). Inhibition of osteoclast differentiation by 1.25-D and the calcimimetic KP2326 reveals 1.25-D resistance in advanced CKD. J. Bone Miner. Res..

[20] Kalatzis V., Nevo N., Cherqui S., Gasnier B., Antignac C. (2004). Molecular pathogenesis of cystinosis: Effect of CTNS mutations on the transport activity and subcellular localization of cystinosin. Hum. Mol. Genet..

[21] Topaloglu R., Gulhan B., İnözü M., Canpolat N., Yilmaz A., Noyan A., Dursun İ., Gökçe İ., Gürgöze M.K., Akinci N. (2017). The Clinical and Mutational Spectrum of Turkish Patients with Cystinosis. Clin. J. Am. Soc. Nephrol..

[22] Servais A., Morinière V., Grünfeld J.-P., Noël L.-H., Goujon J.-M., Chadefaux-Vekemans B., Antignac C. (2008). Late-onset nephropathic cystinosis: Clinical presentation, outcome, and genotyping. Clin. J. Am. Soc. Nephrol..

[23] Kiehntopf M., Schickel J., von der Gönne B., Koch H.G., Superti-Furga A., Steinmann B., Deufel T., Harms E. (2002). Analysis of the CTNS gene in patients of German and Swiss origin with nephropathic cystinosis. Hum. Mutat..

[24] Kalatzis V., Cherqui S., Jean G., Cordier B., Cochat P., Broyer M., Antignac C. (2001). Characterization of a putative founder mutation that accounts for the high incidence of cystinosis in Brittany. J. Am. Soc. Nephrol..

[25] Soliman N.A., Elmonem M.A., van den Heuvel L., Abdel Hamid R.H., Gamal M., Bongaers I., Marie S., Levtchenko E. (2014). Mutational Spectrum of the CTNS Gene in Egyptian Patients with Nephropathic Cystinosis. JIMD Rep..

[26] Shotelersuk V., Larson D., Anikster Y., McDowell G., Lemons R., Bernardini I., Guo J., Thoene J., Gahl W.A. (1998). CTNS mutations in an American-based population of cystinosis patients. Am. J. Hum. Genet..

[27] Gaillard S., Roche L., Lemoine S., Deschênes G., Morin D., Vianey-Saban C., Acquaviva-Bourdain C., Ranchin B., Bacchetta J., Kassai B. (2021). Adherence to cysteamine in nephropathic cystinosis: A unique electronic monitoring experience for a better understanding. A prospective cohort study: CrYSTobs. Pediatr. Nephrol..

[28] Andrzejewska Z., Nevo N., Thomas L., Chhuon C., Bailleux A., Chauvet V., Courtoy P.J., Chol M., Guerrera I.C., Antignac C. (2016). Cystinosin is a Component of the Vacuolar H+-ATPase-Ragulator-Rag Complex Controlling Mammalian Target of Rapamycin Complex 1 Signaling. J. Am. Soc. Nephrol..

